# Investigation of dacomitinib on reducing cell necrosis and enhancing cell apoptosis in C6 glioma rat model by MRI

**DOI:** 10.1042/BSR20190006

**Published:** 2019-03-06

**Authors:** Wang-Sheng Chen, Lan Hong, Fei Wang, Jian-Jun Li

**Affiliations:** 1 Department of Radiology, Hainan General Hospital/Hainan Hospital of Hainan Medical University, Haikou 570311, Hainan Province, China; 2 Department of Gynecology, Hainan General Hospital/Hainan Hospital of Hainan Medical University, Haikou 570311, Hainan Province, China

**Keywords:** Cho/Cr ratio, Dacomitinib, glioma, Immunohistochemistry, necrotic

## Abstract

Background: Glioma is one of the most epidemic and obstinate types of cancer in the central nervous system (CNS) with poor survival rate. Dacomitinib inhibited cell viability and proliferation of epidermal growth factor receptor (*EGFR*)-amplified glioma. In the present study, the regional effects of Dacomitinib on tumor necrosis was investigated. Methods: A C6 rat glioma model was evaluated using proton magnetic resonance spectroscopy (^1^H-MRS), diffusion weighted imaging (DWI), and morphological T2-weighted imaging (T2W). The effects of Dacomitinib on glioma cells were investigated using methods of immunohistochemistry and Hematoxylin and Eosin (H&E) staining. Results: The obtained data indicated that metabolite ratios were significantly decreased (all *P*<0.05) in the Dacomitinib-treated group compared with C6 glioma control group. The ADC value of necrotic core in Dacomitinib group was significantly lower than that in control group. In addition, the expression of Ki-67 in Dacomitinib-treated group (50.32 ± 5.61) was significantly lower than that in control group (*P*<0.05). The apoptotic index (AI) (28.01 ± 2.37) in Dacomitinib-treated group was significantly higher than that in control group (11.58 ± 3.17). Conclusion: The results demonstrated that the Dacomitinib could suppress glioma cell necrosis and proliferation.

## Introduction

Glioma is one of the most common malignant tumors in the central nervous system (CNS) [1]. Although multiple treatments such as surgery, radiotherapy, and temozolomide chemotherapy have been used, the survival rate of glioma patients is still extremely low. Classical glioma is characterized by amplification of epidermal growth factor receptor (*EGFR*) gene, which leads to EGFR protein overexpression [2]. Therefore, to identify more effective therapeutic targets, it is essential to understand the biochemical and molecular pathways that control invasion of glioma cells ahead. Wnt/β-catenin signaling is implicated in glioma development and contributes to clinical malignancy grade and worse prognosis of glioma patients [3–5]. However, more effective treatment options are still required to discover to release or cure the symptoms of glioma patients.

Dacomitinib (PF299804, Pfizer) is a second-generation, oral and irreversible pan-HER tyrosine kinase inhibitor, which exhibits activity on Erlotinib and Gefitinib-resistant non-small cell lung cancer (NSCLC) in non-clinical models [6]. A recent multicenter phase II trial has demonstrated Dacomitinib has a limited single-agent activity in recurrent GB with EGFR amplification with or without variant III (EGFRvIII) deletion [6]. Systemic administration of Dacomitinib could effectively block EGFR signal transduction *in vivo* and affect the growth and survival of EGFR-amplified glioma cells [7]. Two phase II clinical trials on recurrent EGFR-amplified glioma have been carried out (NCT01520870 and NCT01112527) [7]. Dacomitinib has been proved to be effective in some glioma cell lines and U87 xenografts [8]. Despite these findings, the roles of Dacomitinib in different tumor regions have not been extensively clarified yet. For example, the effects of Dacomitinib on necrosis and cell proliferation region have not been reported till now. Proton magnetic resonance spectroscopy (^1^H-MRS) is a non-invasive imaging method for detecting metabolite signals, including choline compounds (Cho), N-acetylaspartate (NAA), creatine (Cr), lactate (Lac) and mobile lipid (Lip) [9,10]. The purpose of this study was to evaluate whether Dacomitinib had effects on cell necrosis and cell proliferation in C6 glioma rat model by using diffusion weighted imaging (DWI) and ^1^H-MRS. Histology, immunohistochemistry, and microarray analyses were performed to observe cell necrosis, cell proliferation, and apoptosis. The results of the present study would facilitate clinical trials of Dacomitinib in glioma patients and suggest potential synergistic approaches and predictive markers.

## Materials and methods

### Cell culture and C6 glioma rat model

C6 glioma cells (Institute of Biochemistry and Cell Biology, SIBS, CAS) were cultured in DMEM at 37°C in a humidified atmosphere of 5% CO_2_. C6 rat glioma cells were implanted according to the previous method [11]. A total of 1 × 10^7^ C6 cells were implanted into male pathogen-free Sprague–Dawley (SD) rats (180–220 g) using a stereotactic device (Cayunga, Kopf, California). Rats were purchased from Shanghai Laboratory Animal Center (CAS, Shanghai, China) and were group housed in ventilated cages in a 12-h light/dark photocycle with food and water *ad libitum*.

### Dacomitinib treatment

Rats were given Dacomitinib (Ryss Laboratory, Union City, California) (0.018% w/v) by drinking water. Rats were divided into two groups, including Dacomitinib group (7.5 mg/kg/day Dacomitinib by oral gavage for 7 days) and control group (0.5% w/w hydroxypropyl-methylcellulose). Dacomitinib was administered when the tumor volume reached 10–15 mm^3^ at 15 days after glioma cell implantation until the end of the study. Rats in untreatment (UT) group were given normal drinking water. The daily dosage of Dacomitinib was measured by weighing water bottles for each rat in a separate cage. No significant differences were observed in the liquid intake of the compounds amongst these rats (the average daily intake of Dacomitinib was approximately 10 mg/kg/day). Rats were anesthetized with 1.5% isoflurane before and during MR examination. The study was performed in strict accordance with the guidelines adhered to the Guide for the Care and Use of Laboratory Animals (8th edition, National Academies Press), and the protocol was approved by the Committee on the Ethics of Animal Experiments at the Hainan Hospital of Hainan Medical University/Hainan General Hospital (approval number HGH2014031).

### MRI scanning

^1^H-MRS was used in the present study. The ^1^H-MRS values of C6 glioma rat model treated with UT (*n*=14) or Dacomitinib (*n*=9) and 12 SD rats were obtained. MRI scanning was performed on a 3.0 T MRI system (Discovery 750w, GE Healthcare, Milwaukee, Wisconsin). The system has 70 mm orthogonal volume coil for signal transmission and head surface coil for signal reception, active-passive gradient shielding (maximum 200 mT/m, 2 mT/m/A), and a water heater to maintain body temperature at 37°C. T2-weighted axial fast spin echo images (FSE T2WI, TR/TE, 4826 ms/120 ms; matrix size = 256 × 256) and T1-weighted images (TR/TE = 2422.1 ms/24 ms) were performed with a field of view of 6.0 cm and slice thickness of 2.0 mm. The ^1^H-MRS values were collected by stimulated echo acquisition mode (STEAM), TE = 4.2 ms, TM (mixing time) = 10 ms, T = 3000 ms, 256 average values, spectral width = 4012 Hz, and each scan took 10 min and 55 s. Eddy current correction was used to maximize signal intensity and reduce peak linewidth, and non-suppressive MR spectra were pre-collected. Water was suppressed by variable power radio frequency pulses and optimized relaxation delay (VAPOR). In all cases, local oscillation (Fastmap, Bruker Biospin) was performed by using first-order and second-order adjustments. The peak width of the water peak (full width at half maximum) was less than 30 Hz. A total of 3.0 × 3.0 × 3.0 mm^3^ stereo pixels were located in the tumors or contralateral normal brain tissues, and the number of tumors in the stereo pixels was maximized.

Omni-Kinetics software (GE Healthcare) was used to analyze MRS data. Image scaling was performed by calibrating water peak (4.78 ppm) and taking ppm as the unit. In the tumor tissue, the main brain metabolic peaks were identified as NAA (2.05 ppm), choline and choline-containing compounds (total Cho) (3.22 ppm), Cr and Cr phosphate (total Cr) (3.03 ppm), as well as mobile lipids (which can not be found in normal brain tissue). Mobile lipids were detected in the tumor tissue of 1.6 ppm of methylene and 0.8 ppm of fatty acyl-methyl. Quantitative evaluation of metabolites was performed to calculate the peak area ratio of tumor metabolites to contralateral choline and creatine: Cr/Cho, NAA/Cho, Lip 1.3 (methylene)/Cho, Lip 0.9 (methyl)/Cho, Cho/Cr, NAA/Cr, Lip 1.3/Cr and Lip 0.9/Cr.

### Quantitative analysis of tumor necrosis

The percentages of tumor necrosis in UT group (*n*=10) and Dacomitinib group (*n*=8) were calculated according to T2-weighted imaging (T2W). Tumor necrosis volume (mm^3^) was measured by the area of hand-painted ROIs, multiplied by the thickness and number of sections at the core of the tumors and necrotic tumors. The formula for calculating the percentage of tumor necrosis is as follows: (total volume of necrotic tumor/total volume of tumor) × 100% as previously reported [12].

### Quantitative analysis of the number of tumor cells

AprioScanScope image analysis was used in the present study. A representative slide of Hematoxylin and Eosin (H&E) staining was selected from each group (UT group and Dacomitinib group) to calculate the density of tumor cells. A total of 3 × 1 mm^2^ ROIs in each H&E slide were taken to count the number of tumor cells. The cells adjacent to or located in the necrotic or inflammatory areas were excluded when counting tumor cells. The number of cells per mm^2^ was measured.

### Histological and immunohistochemical analyses

As previously reported [13], the tissues were fixed with formalin, embedded in paraffin, sectioned, and stained with H&E. Immunohistochemical analysis were performed for proliferation (Ki-67, 1:500 dilution; MIB-1, 1:800 dilution; Dako, Glostrup, Denmark) and apoptosis (caspase-3, 1:500 dilution, Affinity Biosciences, Cincinnati, OH, U.S.A.) using the Leica BondIII autostainer (Leica Microsystems, Mount Waverley, Victoria, Australia) according to the manufacturer’s protocol.

### Statistical analysis

SPSS Statistics software 22.0 version was used for statistical analysis (Chicago, Illinois, U.S.A.). Peak area ratio, ADC value, total necrosis percentage, tumor cell density, Ki-67LI, and apoptotic index (AI) were expressed as (mean ± S.D.). Student’s *t* test was used to compare the differences between two groups. A *P*-value <0.05 was considered as statistically significant difference.

## Results

### Dacomitinib inhibited tumor necrosis in C6 glioma rat model

**Figure 1:**
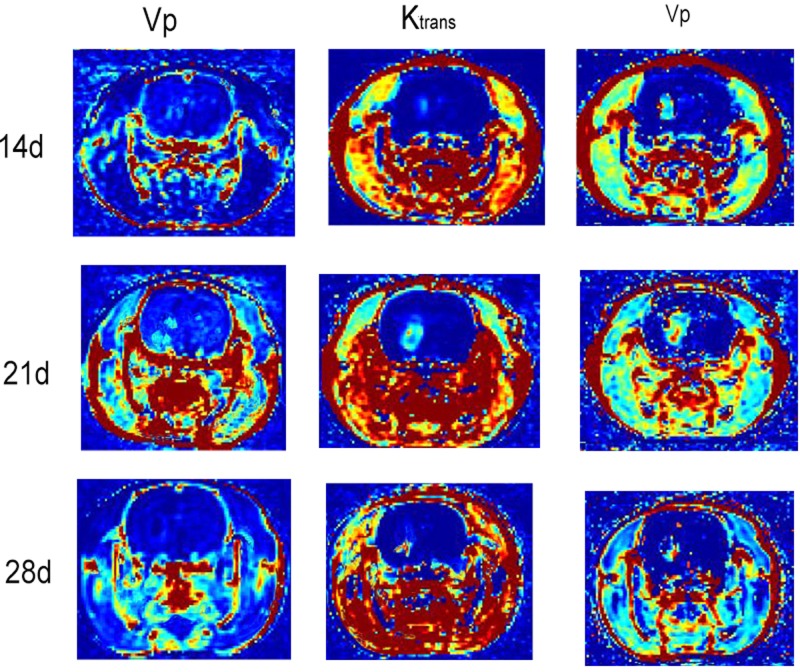
Permeability tissue slices at three time points were shown by typical ^1^H-MR images after C6 glioma model establishment Spectra were obtained at the end of tumor growth. The spectral intensity of untreated tumors was the same as that of Dacomitinib group and normal brain.

As shown in Figure 1, the transplanted tumors demonstrated nodules or masses. T1WI images displayed low signal of tumors while T2WI images exhibited high signal of tumors. Necrosis was observed at 3 weeks after transplantation.

**Figure 2:**
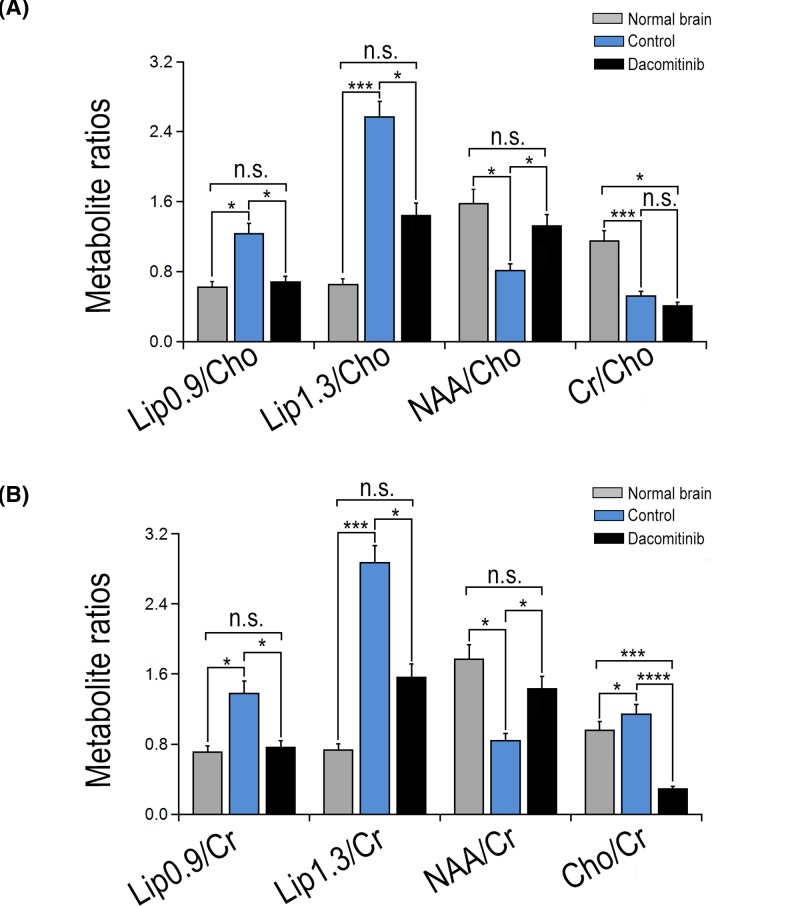
The effect of Dacomitinib on the metabolite ratios of C6 glioma The ratio of brain metabolites in normal rat brain and C6 rat glioma treated with Dacomitinib was compared with Cho (**A**) or Cr (**B**) in contralateral (normal) brain. The ratios increased significantly in the control group compared with that in the normal brain. The values are expressed as mean ± S.D. *, *P*<0.05; ***, *P*<0.001; n.s., non-significant.

As shown in Figure 2, at the end of tumor progression the metabolite ratios, including Lip 0.9/Cho (*P*=0.017), Lip 0.9/Cr (*P*=0.017), Lip 1.3/Cho (*P*=0.027), and Lip 1.3/Cr (*P*=0.023) in Dacomitinib group were significantly lower than those in UT group.

**Figure 3:**
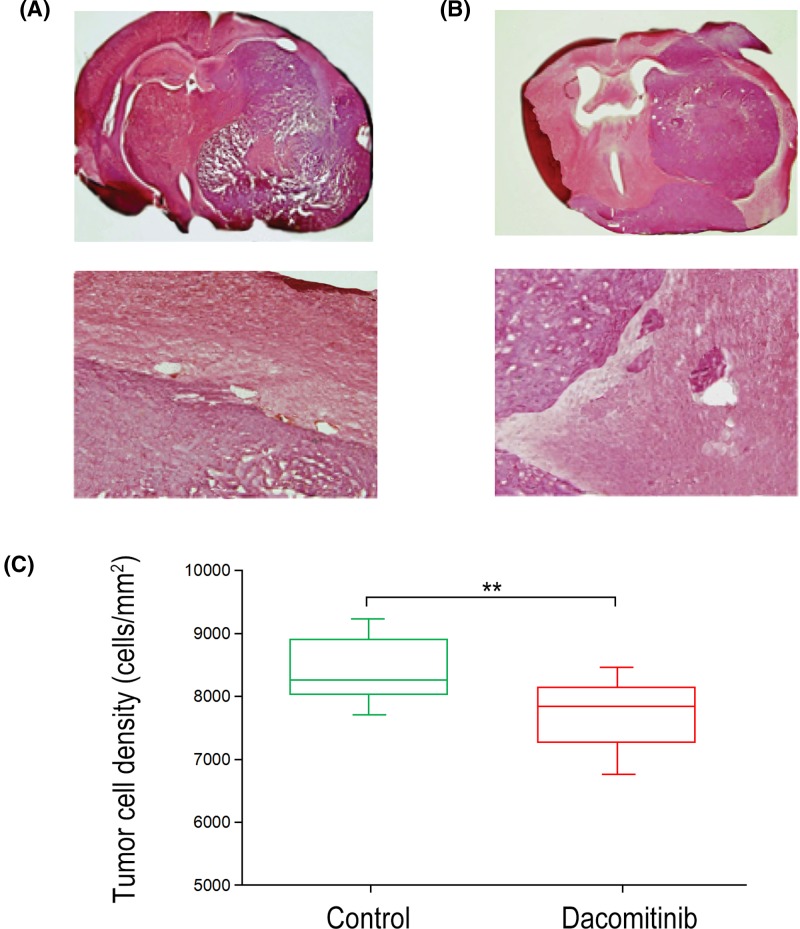
Histological evaluation of Dacomitinib’s effects on C6 glioma rat Typical H&E sections of C6 glioma in the control group (**A**) and Dacomitinib group (**B**) were selected. (**C**) The density of tumor cells in the control group was higher than that in Dacomitinib group. **, *P*<0.01.

**Figure 4:**
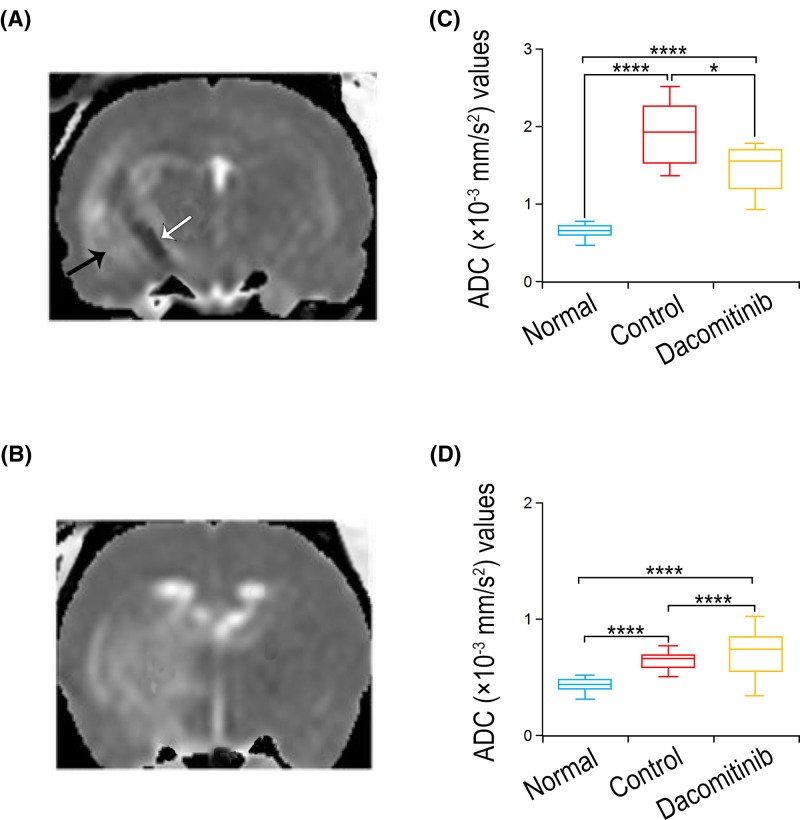
The ADC regional changes in C6 glioma after Dacomitinib treatment The representative ADC of C6 glioma rats in the control group (**A**) or Dacomitinib group (**B**). (**C**) The ADC value of necrotic core in Dacomitinib group was significantly lower than that in the control group, but both were higher than that in the normal brain. (**D**) The ADC value in the non-necrotic parenchymal area of tumors in Dacomitinib group was significantly higher than that in the control group (*n*=12, >1600 ROIs), but both were higher than that in the normal brain. *, *P*<0.05; ****, *P*<0.0001.

Histological observations of the periphery and necrotic core of the tumors were shown in Figure 3. ADC was generated from histological data (Figure 4A,B) and was used to determine the ADC values of necrotic core of tumors in each group. The ADC value of necrotic core in Dacomitinib group was significantly lower than that in UT group (*P*<0.05), both of which were significantly higher than that in control group (*P*<0.0001) (Figure 4C).

**Figure 5:**
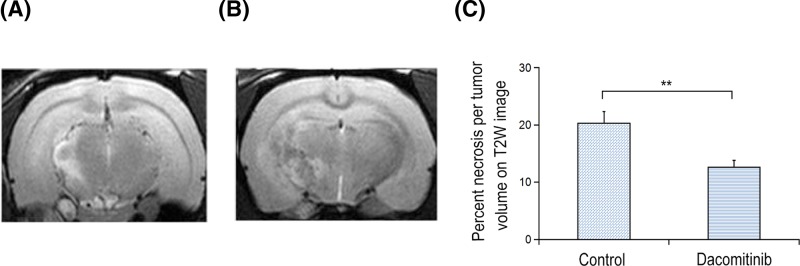
Representive T2W images of C6 glima rats treated with Dacomitinib Representative T2W images of C6 glioma rats in the control group (**A**) or Dacomitinib group (**B**). The dashed line represents the necrotic core area and the dotted line represents the non-necrotic parenchymal area of tumors. (**C**) The volume percentage of necrotic tumors in the control group was significantly higher than that in Dacomitinib group. **, *P*<0.01.

The percentage of tumor necrosis in UT group (20.08 ± 2.00%, *n*=10) was significantly higher than that in Dacomitinib group (11.85 ± 1.20%, *n*=7) (Figure 5). The volume of tumor in Dacomitinib group was 232.42 ± 87.46, which was significantly larger than that in UT group (123.75 ± 62.38) (*P*=0.021).

Matrix Gla protein (MGP) and microfibrillar-associated protein 4 (MFAP4) are directly related to calcium channels regulation, and both their expressions were down-regulated by Dacomitinib. Dacomitinib treatment could down-regulate MGP and MFAP4 expression by 3.17- and 2.14-fold, respectively. Two other genes related to calcium channels regulation were also down-regulated by Dacomitinib, such as fibronectin type III domain containing protein 1 gene, which is associated with G protein βγ, and ADAM metallopeptidase, which is associated with tumor necrosis factor (TNF).

### Dacomitinib inhibited cell proliferation in C6 glioma rats

**Figure 6:**
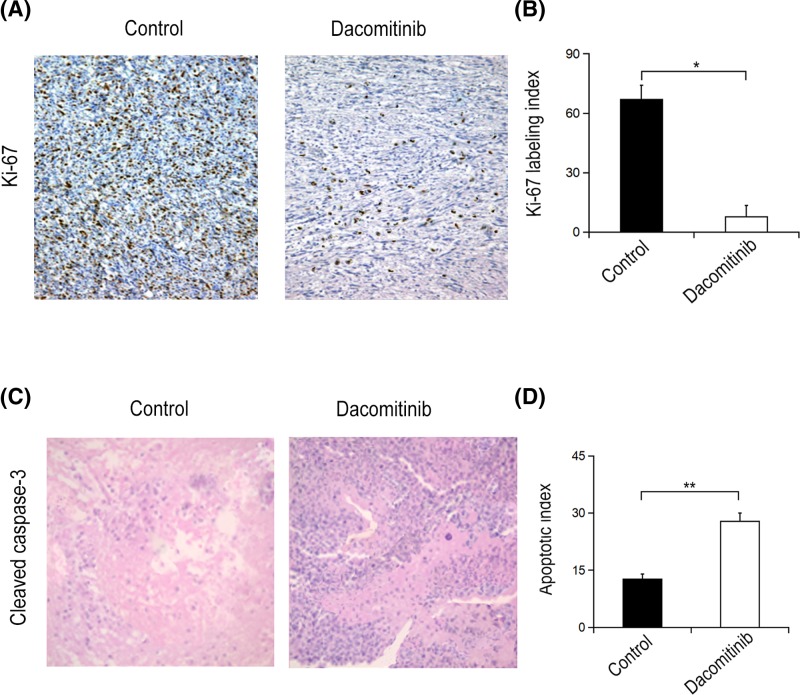
Immunohistochemical stainning of Ki-67 in C6 glioma rats treated with Dacomitinib Immunohistochemical staining of Ki-67 in nucleus of C6 glioma rats in UT group (×200) and Dacomitinib group (**A**) (×200). (**B**) The Ki-67 marker in Dacomitinib group was significantly lower than that in the control group. Immunohistochemical staining of activated caspase-3 nucleus of C6 glioma rats in the control group (×200) and Dacomitinib group (**C**) (×200). (**D**) The AI in Dacomitinib group was significantly higher compared with that in the control group. *, *P*<0.05; **, *P*<0.01.

^1^H-MRS data showed that the ratio of Cho/Cr of rats treated with Dacomitinib was significantly lower than that of C6 glioma rats treated with UT (*P*=0.033) (Figure 2B). Tumor sections stained with H&E were used to measure the density of tumor cells (number of tumor cells/mm^2^) in both groups. Compared with UT group (8435 ± 129), the cell density of Dacomitinib group decreased significantly (7583 ± 261) (*P*<0.001) (Figure 3). DWI showed that the ADC value of non-necrotic tumor parenchyma in Dacomitinib group was significantly higher compared with UT group (*P*<0.05) (Figure 4D). Proliferation of tumor cells was assessed by the expression of Ki-67. The expression of Ki-67 in rats treated with Dacomitinib (50.32 ± 5.61) was significantly lower than that in UT group (70.82 ± 6.03) (*P*<0.05, Figure 6A–C). Dacomitinib increased cell apoptosis. AI (28.01 ± 2.37) in Dacomitinib group was significantly higher than that in UT group (11.58 ± 3.17) (*P*<0.01, Figure 6D).

## Discussion

The present study evaluated the effect of Dacomitinib on the heterogeneous necrotic core and non-necrotic parenchymal region of C6 glioma. Studies found that Dacomitinib could inhibit tumor necrosis and cell proliferation in glioma [14]. In our study, ^1^H-MRS showed that the ratios of Lip1.3/Cr, Lip1.3/Cho, Lip0.9/Cr, and Lip0.9/Cho in Dacomitinib group were lower than those in UT group, which may be related to the suppression of necrosis.

The role of mobile lipids in cancer has attracted abundant attention from reviews and articles [15]. Increased levels of lipid are indispensably associated with necrosis in patients with brain tumors, and lipids are considered to be reliable biomarkers for the diagnosis and monitoring of therapeutic responses [16,17]. The present study found that AI of rats treated in Dacomitinib group was higher than that in control group, indicating that Dacomitinib also affected apoptosis. Abnormal cell survival and resistance to apoptosis could lead to central necrosis, which is a characteristic of glioma [18]. Glioma tends to produce necrosis, which can drive necrosis as an alternative to cell death, partially because of its resistance to apoptosis.

DWI and histopathological data also confirmed the spectral results. The ADC value in necrotic tumor core of rats treated with Dacomitinib was lower than that of UT group. The inhibitory effect of Dacomitinib on necrotic tumor core of C6 glioma was observed on T2WI images in each group. The percentage of necrotic tumor volume in rats treated with Dacomitinib was lower compared with UT group, probably due to the fact that Dacomitinib affected necrosis by down-regulating genes related to Ca^2+^ channel signaling, thus reducing intracellular Ca^2+^. Microarray results also confirmed that Dacomitinib affected genes associated with Ca^2+^ channel signaling. Dacomitinib down-regulated the expression of MGP and MFAP-4, which were directly related to Ca^2+^ channel signaling. In addition, the expression of other genes related to Ca^2+^ channel signaling also seemed to be down-regulated by Dacomitinib. Since TNF-α is associated with Ca^2+^ channel related genes, the down-regulation of Ca^2+^-related genes may also down-regulate TNF-α expression, which in turn down-regulate NF-κB signaling pathway, thereby reducing cell necrosis. The expression of ADAMTS8 directly related to TNF-α was also down-regulated.

Furthermore, it was found that Dacomitinib could inhibit cell proliferation of C6 glioma rats. Previous studies have confirmed that the relative increase in choline in most well-differentiated glioma depended on the increase in membrane synthesis and acceleration of cell proliferation [19]. Total choline is a complex peak consisting of choline, choline phosphate, and choline glycerophosphate [20]. The increase in total choline in tumors is mainly due to the synthesis and accumulation of choline phosphate, which is a metabolite produced by rapid uptake and phosphorylation of choline. It is an essential substance for downstream synthesis of phosphatidylcholine and a major membrane phospholipid, accounting for approximately 25% of lipid of mammalian cells. The data in the present study also indicated that Dacomitinib reduced the ratio of Cho/Cr at the end of tumor progression due to reducing tumor cell proliferation. DWI and histopathological data consistently confirmed this finding.

Intracellular and extracellular spaces and their exchange are helpful to measure ADC. With the increase in cell density, the curvature of extracellular migration pathway increases, which can reduce water migration, thus reducing the ADC value. Based on this finding, ADC can be used as an indicator of relative cell density in certain tissue or cell types, such as the change of tumors over time after treatment. ADC is inversely proportional to cell density. After effective treatment, the cell density decreased and diffusivity increased due to cell apoptosis and necrosis [21]. The increase in ADC indicated a positive response to treatment. Similarly, the increase in water ADC after treatment was directly related to the number of dead tumor cells, and was attributed to the release of water into extracellular space [22]. It was found that the ADC value in the parenchyma of non-necrotic tumors treated with Dacomitinib was significantly higher than that of control group, manifesting that Dacomitinib restricted spread of tumor cells and resulted in the decrease in tumor cell population during treatment of C6 glioma. H&E staining of tumor sections and Ki-67 expression were used to evaluate tumor cell density and proliferation, respectively. Compared with control group, the cell density and Ki-67 LI expression in Dacomitinib group were significantly decreased. The results of the present study were consistent with previous studies, confirming the positive correlation between Cho signaling and cell density, and the inverse correlation between cell density and ADC value in glioma.

## Conclusion

In summary, the acquired data from the present study indicated that Dacomitinib could suppress necrosis and proliferation of tumor cells. ADC and spectral choline measurements were related to cell density of C6 glioma. *In vivo* studies found that Dacomitinib played multiple roles in different regions of C6 glioma. All these results further supported the potential application of Dacomitinib as a potential antitumor drug.

## Availability of data and materials

The datasets generated and/or analyzed during the current study were available from the corresponding author on reasonable request.

## Abbreviations

ADAM: a disintergrin and metalloprotease
ADAMTS8: a disintergrin and metalloprotease with thrombospondin motifs 8
ADC: apparent diffusion coefficient
AI: apoptotic index
Cho: choline compound
Cr: creatine
DMEM: Delbecco’s Modified Eagle’s Medium
DWI: diffusion weighted imaging
EGFR: epidermal growth factor receptor
^1^H-MRS: proton magnetic resonance spectroscopy
H&E: Hematoxylin and Eosin
MFAP4: microfibrillar-associated protein 4
MGP: matrix Gla protein
MR: magnetic resonance
NAA: N-acetylaspartate
NF-kB: nuclear factor kB
ROI: region of interest
SD: Sprague–Dawley
TNF: tumor necrosis factor
T2W: T2-weighted imaging
UT: untreatment
